# A Single Point Mutation in the Mumps V Protein Alters Targeting of the Cellular STAT Pathways Resulting in Virus Attenuation

**DOI:** 10.3390/v11111016

**Published:** 2019-11-01

**Authors:** Tahir Malik, Laurie Ngo, Trent Bosma, Steven Rubin

**Affiliations:** 1 DVP/Office of Vaccines Research and Review, Center for Biologics Evaluation and Research, Food and Drug Administration, Silver Spring, MD 20993, USA; 2 GlaxoSmithKline, 14200 Shady Grove Rd, Rockville, MD 20850, USA

**Keywords:** Mumps, non-segmented, negative-stranded, enveloped, RNA virus, paramyxovirus, STAT, neurovirulence

## Abstract

Mumps virus (MuV) is a neurotropic non-segmented, negative-stranded, enveloped RNA virus in the *Paramyxovirus* family. The 15.4 kb genome encodes seven genes, including the V/P, which encodes, among other proteins, the V protein. The MuV V protein has been shown to target the cellular signal transducer and activator of transcription proteins STAT1 and STAT3 for proteasome-mediated degradation. While MuV V protein targeting of STAT1 is generally accepted as a means of limiting innate antiviral responses, the consequence of V protein targeting of STAT3 is less clear. Further, since the MuV V protein targets both STAT1 and STAT3, specifically investigating viral antagonism of STAT3 targeting is challenging. However, a previous study reported that a single amino acid substitution in the MuV V protein (E95D) inhibits targeting of STAT3, but not STAT1. This provided us with a unique opportunity to examine the specific role of STAT 3 in MuV virulence in an in vivo model. Here, using a clone of a wild type MuV strain expressing the E95D mutant V protein, we present data linking inhibition of STAT3 targeting with the accelerated clearance of the virus and reduced neurovirulence in vivo, suggesting its role in promoting antiviral responses. These data suggest a rational approach to virus attenuation that could be exploited for future vaccine development.

## 1. Introduction

Mumps virus (MuV), a rubulavirus of the *Paramyxoviridae* family, causes an acute infectious disease mainly in children and young adults. In the pre-vaccine era, nearly everyone had been exposed to the virus by 15 years of age based on the presence of virus-specific antibody [1]. The disease, largely under control in countries with national vaccine programs that include use of live, attenuated mumps-containing vaccines, has reemerged over the last decade, even in highly vaccinated populations [2,3,4,5,6,7,8]. The most prominent clinical manifestation of mumps is parotitis; however, more serious complications can occur, including orchitis, meningitis and deafness [9]. The virus is highly neurotropic with evidence of central nervous system (CNS) invasion in more than half of patients with clinical mumps [10,11,12]. The neurotropism of the virus has been a problem for vaccine development. Most mumps vaccines have been causally-linked to aseptic meningitis in vaccinees, highlighting the difficulty in successfully attenuating the virus [13,14]. Use of these vaccine strains have largely been replaced with the highly attenuated Jeryl Lynn vaccine strain; however, the resurgence of mumps despite very good vaccine coverage suggests the need for the development of new, more effective safe vaccines. With the advent of reverse genetics systems for MuV and the ability to assess MuV neurotoxicity in a meaningful animal model, efforts are now underway to identify rational approaches to MuV neuroattenuation [15,16,17,18,19,20,21].

The MuV genome encodes three core proteins: The nucleocapsid protein (N), the phosphoprotein (P), and the large protein (L); two surface glycoproteins: The fusion (F) and the hemagglutinin-neuraminidase (HN) proteins; and the membrane-associated matrix (M) and small hydrophobic (SH) proteins [22]. The gene that encodes the P protein also encodes the V protein and the putative I protein [23,24,25,26].

The MuV V protein is 225 amino acids in length and is likely a structural protein based on reports of its detection in virions and isolated virus lipids [26,27]. While the V open reading frame has been identified in all MuV isolates, expression of the gene is not required for growth in tissue culture or in vivo, as demonstrated by Xu et al. who engineered a recombinant MuV that does not produce a V protein [28,29]. Nonetheless, this protein appears to play an important role in infection, given its ability to bind to cellular signal transducer and activator of transcription (STAT) proteins and targeting them for degradation [30,31]. The MuV V protein can bind to STAT1, STAT2 and STAT3; however, only STAT1 and STAT3 appear to be targeted for destruction [28,32,33,34]. It is generally accepted that STAT1 and STAT2 are antiviral mediators, and while it is presumed that the degradation of STAT3 by the MuV V protein would lead to the inhibition of host antiviral responses allowing the virus to evade the host immune response, in vivo studies to demonstrate this have yet to be performed. The V protein-deficient MuV generated by Xu et al. was found to be highly attenuated in vivo relative to its wild type parent virus (28); however, given that the V protein targets both STAT1 and STAT3, it is not known if this effect was mediated by one, the other, or both STATs. In 2007, Puri et al. showed that a single amino acid change at position 95 in the MuV V protein, from a glutamic acid to an aspartic acid (E95D), disrupts its ability to associate with STAT3, but not STAT1 [34]. However, given the lack of an appropriate animal model, the biological consequences of abrogating STAT3 targeting could not be examined. Here, we have extended these studies and examined the role of STAT3 per se in MuV infection in vitro and in vivo.

## 2. Materials and Methods 

### 2.1. Cell Lines and Primary Cells

Vero cells and FaDu cells (human epithelial cells derived from a squamous cell carcinoma of the pharynx (ATCC, Manassas, VA, USA)) were maintained in Dulbecco’s modified Eagle’s medium (D-MEM) supplemented with 10% fetal calf serum (growth media). 293-A cells (Thermo Fisher Scientific, Waltham, MA, USA) were maintained in growth media supplemented with 0.1 mM MEM Non-Essential Amino Acids (NEAA) (Thermo Fisher Scientific). BHK-BSR-T7/5 cells [16,35], kindly provided by K. Conzelmann, (Munich, Germany), were maintained in growth media and 1 mg/mL neomycin. 

### 2.2. RT-PCR and Sequencing

Reverse transcription (RT) reactions were performed with gene-specific primers and Superscript II reverse transcriptase (Thermo Fisher Scientific) in accordance with the manufacturer’s protocol. All PCR was performed with the Pfx (Thermo Fisher Scientific) or the Expand High Fidelity (Roche Diagnostics Corporation, Indianapolis, IN, USA) enzymes. 

Conventional sequencing: PCR fragments and plasmids were sequenced on an ABI 3100 automated capillary DNA sequencer. Sequence data were analyzed with the Chromas (Technelysium Pty Ltd., Tewantin, Australia) and Jellyfish (LabVelocity, Inc., Los Angeles, CA, USA) software packages. All primer sequences are available upon request. 

Hi-Seq sequencing of viral genomes: 161 µL of virus-containing cell culture supernatants were treated for 2 h at 37 °C with 11,400 gel units of micrococcal nuclease (New England Biolabs, Ipswich, MA, USA), followed by viral RNA extraction using the QIAamp MinElute Virus spin kit (Qiagen, Germantown, MD, USA). The extracted RNA was processed following the protocol for the Illumina TruSeq Stranded mRNA Preparation Kit (Illumina, San Diego, CA, USA), but without the polyA enrichment step. Briefly, approximately 100 ng of RNA was chemically fragmented and reverse-transcribed into cDNAs. Double strand cDNAs were adenylated at the 3′ends and individually indexed, followed by limited-cycle (15) amplification, and purification using Agencourt AMPure magnetic beads (Beckman Coulter, Atlanta, GA, USA). After analyzing the cDNA libraries for size and quality using a BioAnalyser (Agilent Technologies Inc., Santa Clara, CA, USA), paired-end sequencing (100 × 2 cycles) of twelve multiplexed RNA samples per lane was carried out on an Illumina HiSeq2500 sequencer. 

### 2.3. Construction of Plasmids

Full-length molecular clones: 88-1961_WT_ and 88-1961_E95D_: Construction of the 88-1961_WT_ infectious cDNA clone p88-1961FL_A271_ (+) is described elsewhere [16]. This clone is referred to here as p88-1961_WT_ and was used in this study as a reagent to introduce a modified V ORF (containing a G to T change at nucleotide position 285 of the V ORF) into the 88-1961 genome, resulting in plasmid p88-1961_E95D_. Jeryl Lynn (JL_WT_) and JL_E95D_: The Jeryl Lynn clone was constructed by sequential ligation of seven synthetic DNA fragments into the pBluescript vector (Agilent Technologies Inc.). The DNA fragments were commercially synthesized (GenScript; Piscataway, NJ, USA) based on Jeryl Lynn sequence previously reported by Lemon et al. [15]. The 5′ terminal DNA fragment contained sequence for a T7 promoter, while the 3′ terminal fragment contained sequences for a Hepatitis Delta Ribozyme and two T7 terminators. This clone is referred to here as pJL_WT_ and is identical in sequence to the Jeryl Lynn infectious clone except for the introduction of a SbfI site between the N and P genes and an AscI site between the HN and L genes, respectively, (Genbank Accession Number: MN172168). The nucleotide changes required to introduce these restriction sites were limited to the intergenic regions between these ORF’s and did not impact the start and end signal sequences. pJL_WT_ was used to introduce a mutation at position 285 of the V ORF into the Jeryl Lynn genome, resulting in plasmid pJL_E95D_. Additional details of the construction of these three plasmids, including primer sequences, are available upon request. 

Expression plasmids: The construction of the plasmids expressing the 88-1961 N, P, and L proteins has been described previously [16]. Plasmids expressing the Jeryl Lynn N, P, and L were similarly cloned into the pTM1 vector. Plasmids expressing either the 88-1961 or Jeryl Lynn N-terminal FLAG-tagged V and V_E95D_ proteins were constructed by cloning the FLAG-V and FLAG-V_E95D_ sequences flanked by the EcoRI and XhoI restriction sites (synthesized by GenScript) into the pCAGGS vector (kindly provided by J. C. de la Torre, La Jolla, CA, USA) [36] at the corresponding restriction sites. A plasmid expressing an N-terminal FLAG-tagged GFP protein was similarly constructed by cloning the commercially synthesized FLAG-GFP sequence (Genscript) into the pCAGGS vector.

### 2.4. Rescue of Recombinant Virus from cDNA

The rescue of r88-1961_WT_ has been described previously [16]. To rescue virus from cDNA plasmids p88-1961_E95D_, pJL_WT_, and pJL_E95D_, respectively, BHK BSR-T7/5 cells were grown to 95% confluency in 6-well dishes in the absence of antibiotics and a total volume of 2 mL growth media and then transfected with a mixture containing Lipofectamine (Thermo Fisher Scientific), the appropriate full-length plasmid and the supporting plasmids expressing the MuV N, P and L proteins, as described previously [16]. When virus-specific cytopathic effects became apparent (between days 5–10), the supernatant from a single well in the 6-well dish was used to inoculate a confluent monolayer of Vero cells in a 225 cm^2^ flask. The virus inoculum was removed 1 h post-infection, the monolayer rinsed once with growth media, and 20 mLs of fresh growth media were added to the flask. The cells were mechanically detached 4 days later and along with the supernatant subjected to a single freeze–thaw cycle, and clarification by centrifugation at 1200 r.p.m. for 10 min. Virus titer’s were determined by plaque assay on Vero cells as described previously [16]. Viral RNA from stocks of each of four independent rescues of each virus was subjected to Hi-Seq sequencing.

### 2.5. In Vitro Growth Kinetics

Cell-lines: Confluent monolayers of Vero or FaDu cells in 12 well plates were inoculated with the virus at a multiplicity of infection (MOI) of 0.05 and 1.0 PFU/cell in growth media. After incubation for 1 h at 37 °C, inocula were removed, and monolayers washed twice in PBS and overlaid with 2 mL growth media. Every 24 h over a 10 day (MOI = 0.05) or 5 day (MOI = 1.0) period, 200 µL of cell culture supernatant was removed from 2 wells per virus treatment and immediately stored at −70 °C until the determination of virus titers by plaque assay as described previously [16]. The removed volumes of medium were replaced with equal volumes of fresh growth media.

### 2.6. In Vivo Characterization of the cDNA Derived 88–1961 Recombinant Viruses

Neurovirulence: To evaluate virus virulence in vivo, the rat neurovirulence test was performed as described previously [37]. Briefly, newborn rats were inoculated intracerebrally with 100 PFU of virus (in a volume of 10 µL) and sacrificed one month later. Brains were removed, immersion fixed in 10% (*w*/*v*) neutral buffered formalin at 4 °C for 4–5 days, and paraffin-embedded. From each brain hemisphere, one 10 µm sagittal section was selected at a standard distance from the anatomical midline and stained with hematoxylin and eosin. The neurovirulence score, which is a measure of the severity of hydrocephalus, was determined by measuring the cross-sectional area of the brain (excluding the cerebellum) and that of the lateral ventricle on tissue sections from each pair of brain hemispheres per rat. Cross-sectional area measurements were performed using Image Pro Plus image analysis software (Media Cybernetics, Silver Spring, MD, USA). The mean ratio (percentage) of these two measurements on each of the two tissue sections per rat brain was assigned as the neurovirulence score for that particular brain. The neurovirulence score for a given virus variant was determined by the mean neurovirulence score for all brains within a treatment group. Any rats showing signs of pain or distress prior to the planned one-month endpoint were humanely euthanized immediately, but included in the analyses. Strict adherence to the National Research Council Guide for the Care and Use of Laboratory Animals was followed, and all studies were conducted under an approved animal use protocol. Of note, given that r88-1961_WT_ has scored consistently (19.34 +/− 1.531) in the neurovirulence test over many years of testing, the Agency’s Institutional Animal Care and Use Committee recommended that historical data be used for future in-vivo studies when this virus is only being used as a control. Historical neurovirulence data is, thus, presented in Figure 2 to represent r88-1961_WT_.

In vivo growth kinetics: To determine the kinetics of virus production in vivo, newborn rats were inoculated intracerebrally with 100 PFU of virus. On days 1–4, 6 and 8 post inoculation, brains were removed from five rats at each time point, immediately placed on dry ice and stored at −70 °C. Each brain was homogenized in E-MEM (20% *w/v*) and subjected to ultrasonic treatment (3 times for 10 s each) and clarification by centrifugation at 10,000 rpm for 5 min. Virus titer was determined by plaque assay on Vero cells [16].

Confirmation of the E95D substitution in r88-1961_E95D_: To confirm the presence of the engineered mutation following virus replication in rat brain, three animals were inoculated with the rescued virus and sacrificed three days later (a time coinciding with peak virus replication). Homogenates of each brain were prepared as described above, and total RNA was extracted with the RNeasy Plus Mini kit (Qiagen) as recommended by the manufacturer. A region encompassing the V ORF was amplified by RT-PCR and the PCR product sequenced using the reverse primer to verify the presence of the mutation.

### 2.7. Assessment of Protein Expression

Infected cell lysates: Vero cells (1 × 10^6^ cells/well of a 6-well plate) were infected at an MOI = 1.0 with each virus. At each harvest time-point, the cells were washed with ice-cold PBS supplemented with 1X Halt^TM^ Protease and Phosphatase Inhibitor Cocktail (Thermo Fisher Scientific) and lysed directly in 200 µL PBS + 200 µL Laemmli sample buffer (Bio-Rad, Hercules, CA, USA) containing 2.5% of freshly added β-mercaptoethanol. Lysates prepared from rat brains, removed 3 days post-infection, were prepared as described above (see the “in vivo growth kinetics” section) and lysed in a 1:1 volume of Laemmli sample buffer containing 2.5% of freshly added β-mercaptoethanol. All samples were stored frozen at −70 °C until use. The lysates were resolved by SDS-PAGE (4–20% TRIS-glycine gels, Lonza Inc., Allendale, NJ, USA) followed by western blotting.

Transfected cell lysates: 293-A cells were grown to 95% confluency in 6-well dishes in the absence of antibiotics and a total volume of 2 mL growth media and then transfected with a mixture containing Lipofectamine and the appropriate plasmid as described previously [16]. At 48 h post-transfection, the cells were processed for immunoprecipitation as described below. A fraction of the cells (1/30th) were washed with ice-cold PBS supplemented with 1X Halt^TM^ Protease and Phosphatase Inhibitor Cocktail, resuspended in PBS and lysed in a 1:1 volume of Laemmli sample buffer containing 2.5% of freshly added β-mercaptoethanol. The samples were stored frozen at −70 °C until use.

Immunoprecipitation: Immunoprecipitation was performed essentially per the FLAG Immunopreciptation Kit (Sigma-Aldrich, St. Louis, MO, USA). Briefly, transfected cells from a single well of a 6 well-plate were washed twice with ice-cold PBS and lysed directly in 500ul ice-cold lysis buffer (50 mM Tris HCL (pH 7.4), 150 mM NaCl, 1 mM EDTA and 1% Triton X-100) supplemented with 1X Halt^TM^ Protease and Phosphatase Inhibitor Cocktail. Following a 15 min incubation at 4 °C on a rocking platform, the supernatants and cellular debris from each lysate were transferred into an Eppendorf tube and centrifuged (4 °C) for 10 min at 12,000 *g*. The supernatants were transferred into a fresh Eppendorf tube and pre-cleared by incubation with mouse IgG agarose for 1 h at 4 °C, followed by incubation with anti-FLAG M2 agarose resin overnight at 4 °C on a rotating mixer to capture FLAG-V or FLAG-V_E95D_ bound complexes. Each resin was washed five times with 1× wash buffer (50 mM Tris HCL (pH 7.4), 150 mM NaCl) followed by incubation and boiling for 3 min with 60 μL of 2× sample buffer (125 mM Tris HCL, (pH 6.8), 4% SDS, 20% glycerol and 0.004% bromophenol blue) to elute the bound proteins. The supernatants were transferred to a fresh tube, and all samples were stored frozen at −70 °C until use. The samples were resolved by SDS-PAGE (4–20% TRIS-glycine gels) followed by western blotting. 

Protein detection and quantification: Protein expression was detected as necessary with antibodies against STAT 1 (C-24; 1/1000) (Santa Cruz Biotechnology, Inc., Dallas, TX, USA), STAT 2 (1/1000) (Abcam, Cambridge, MA, USA), STAT 3 (C-20; 1/1000) (Santa Cruz Biotechnology, Inc.), GFP (1/10,000) (Thermo Fisher Scientific), FLAG (1/500) (Sigma-Aldrich), and DDB1 (1/500) (Abcam). V expression was detected with a rabbit anti-mumps antibody (1/10,000) that recognizes both the P and V proteins (produced in-house). In order to control for the equivalent loading of samples between lanes, actin was detected by an anti-actin antibody (1/5000) (Abcam). Protein bands were visualized by staining with horseradish peroxidase (HRP)-conjugated goat anti-rabbit antibody (1/10000) (Millipore, Billerica, MA, USA) followed by Enhanced Chemi-Luminescence (ECL) (Santa Cruz Biotechnology, Inc.). Quantification of protein band intensity was performed using the Image J software [38] as described by H. Davarinejad (http://www.yorku.ca/yisheng/Internal/Protocols/ImageJ.pdf). Briefly, X-Ray films were scanned at 600dpi, converted to greyscale, saved in a JPEG format and analyzed using Image J. For each image, a region of interest (ROI) was defined for one row of protein at a time, using the rectangle tool from Image J to draw a frame around the largest band in the row. Measurements were recorded for each protein in the row, as well as background measurements directly above each band of interest. This process was repeated for all rows of protein in a given image. Upon completion, all the data was exported to Microsoft Excel for analysis as described in Algorithm 1 below. 

**Algorithm 1:** Calculation of Relative quantification values
**Input:** Pixel densities measured by Image J
**Output:** Relative quantification values expressed as a percentageInvert the value of the pixel densities (X)(255 − X) = YDeduct the matched inverted background valueY – inverted background = Z (net pixel density value)Calculate the ratio of the net pixel value divided by the net loading control valueZ ÷ net loading control = R (Relative quantification value expressed as a percentage)


## 3. Results

### 3.1. Sequence Characterization of r88-1961_E95D_, rJL_WT_ and rJL_E95D_

Mumps virus, like all RNA viruses, exists as a quasi-species; a swarm of highly related genome variants. The first few mumps virus transcripts produced from the cDNA clone are likely free of such heterogeneity; however, upon replication in BHK-BSR-T7 cells, and then a passage in Vero cells to produce working stocks, minor sequence heterogeneity is gradually acquired. Because even a low level of heterogeneity can theoretically impact virus attributes, including attenuation and virulence, we rescued multiple viruses from each full-length cDNA clone and subjected them to high throughput sequencing followed by a base-by-base examination of the sequence. Only rescues lacking heterogeneity of potential significance (i.e., absence of heterogeneity in known conserved domains) and no mutations at the 100% level were used. Importantly, each rescued virus selected for this study was free of mutations and sites of heterogeneity above 5% in P/V, and while we cannot rule out that mutations existing at such low levels could impact virus virulence, it is impractical, if not infeasible to evaluate such mutants on an individual level. The rescued virus stocks were labelled r88-1961_E95D_, rJL_WT and_ rJL_E95D_, respectively.

### 3.2. Characterization of the in vitro Growth Kinetics of r88-1961_E95D_

**Figure 1:**
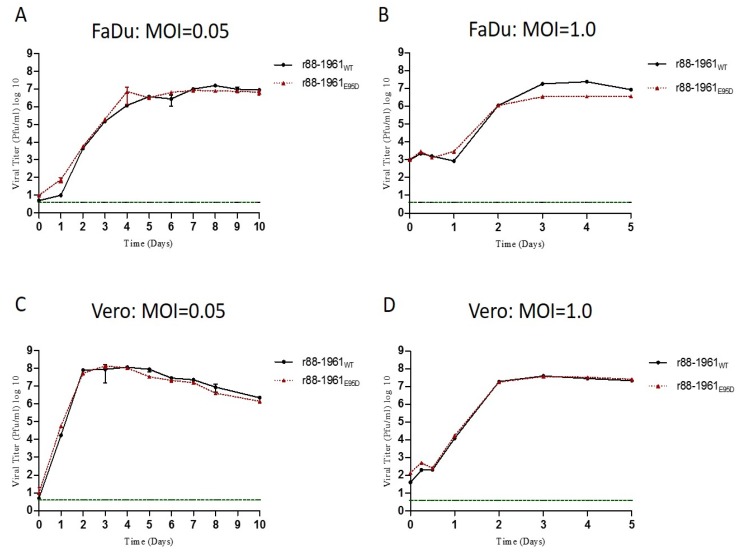
Comparison of the in vitro growth kinetics of r88-1961_WT_ versus r88-1961_E95D_. Confluent monolayers of FaDu (**A**,**B**) and Vero cells (**C**,**D**) were infected with r88-1961_WT_ or r88-1961_E95D_ at an MOI of 0.05 (**A**,**C**) or 1.0 (**B**,**D**), and cumulative virus production was determined. The data represents mean values ± standard error of the mean from two independent experiments. The dashed horizontal line in each graph indicates the limit of detection of the assay. Similar growth kinetics and titers were observed between the two viruses tested in the same culture systems/conditions.

To analyze the in vitro growth characteristics of r88-1961_E95D_ relative to r88-1961_WT_, cumulative virus production was determined for each virus in a disease-relevant human pharynx epithelial-derived immortalized cell-line (FaDu; Figure 1a,b) and in Vero cells (Figure 1c,d) at low and high MOI’s. In FaDu cells, the viruses replicated similarly at a low MOI (*p* = 0.4725, Two-way ANOVA) and within one log of each other at some time points at a high MOI (not statistically significant; *p* = 0.1221, Two-way ANOVA). In Vero cells, the two viruses grew nearly identically at both low *(p* = 0.4321, Two-way ANOVA) and high (*p* = 0.5138, Two-way ANOVA) MOI’s tested.

### 3.3. Characterization of the in vivo Virulence and Growth Kinetics of r88-1961 _E95D_ in the Newborn Rat Model

**Figure 2:**
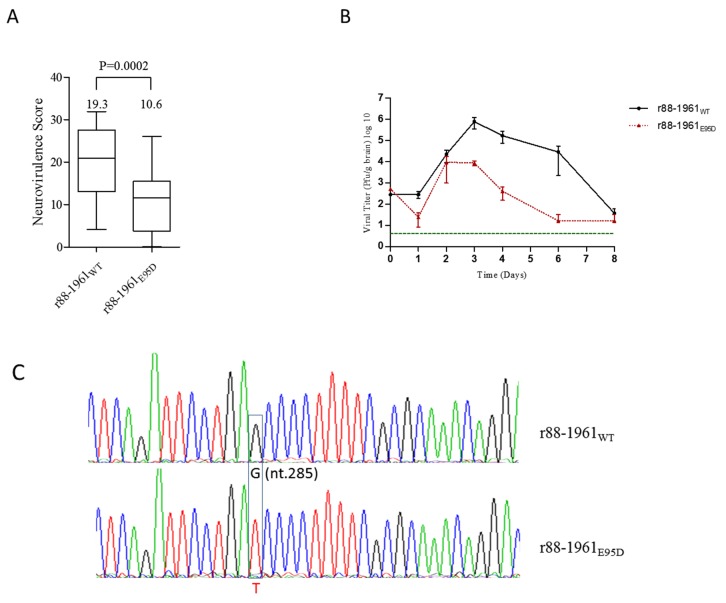
The severity of hydrocephalus and in vivo growth kinetics in rats inoculated with r88-1961_WT_ and r88-1961_E95D_. (**A**) Newborn rats were inoculated i.c. with r88-1961_WT_ (31 animals) and r88-1961_E95D_ (26 animals) and euthanized one month later. The brains were then removed, and hydrocephalus scores calculated. The vertical lines in each box plot represent the maximum and minimum hydrocephalus score values, respectively, for a given virus, while the horizontal lines within each box represent the median scores. The mean scores for each virus are indicated at the top of each plot. (**B**) Newborn rats (5 animals per time point) were inoculated intracerebrally with r88-1961_WT_ and r88-1961_E95D_ and euthanized on days 1–4, 6 and 8 post inoculation. The brains were processed, and viral titer determined. Error bars represent standard error of the mean, and the dashed horizontal line indicates the limit of detection of the assay. (**C**) Maintenance of the wild type V nucleotide (G) in r88-1961_WT_ and the presence of the engineered substitution (T) in r88-1961_E95D_ was confirmed in brain lysates prepared from rats inoculated intracerebrally with r88-1961_WT_ and r88-1961_E95D_ and euthanized on day 3 post inoculation. The sequence was confirmed independently in three rat brains per virus variant.

The virulence of r88-1961_E95D_ relative to r88-1961_WT_ was assessed in vivo using the previously described rat neurovirulence assay [37]. As shown in Figure 2a, there was a moderate, but statistically significant reduction in the neurovirulence scores of rats inoculated with r88-1961_E95D_ (10.6) compared to r88-1961_WT_ (19.3), *p* = 0.0002 (*t*-test).

r88-1961_E95D_ also grew to lower titers in rat brain as compared to r88-1961_WT_ throughout the time course studied (*p* = 0.0178, Two-way ANOVA) (Figure 2b). Additionally, r88-1961_E95D_ rapidly declined in titer after day 3 post inoculation in comparison to r88-1961_WT_, which persisted at high titers for several more days (Figure 2b). Maintenance of the engineered mutation in r88-1961_E95D_ in rat brain was confirmed by extraction of total RNA from brain homogenates at day 3 p.i. (time of maximal virus production), followed by RT-PCR and sequencing (Figure 2c).

### 3.4. STAT Protein Degradation In Vitro and In Vivo 

**Figure 3:**
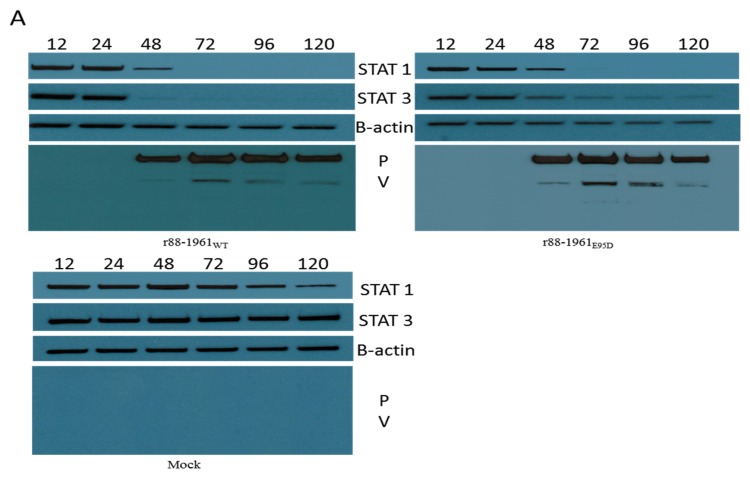

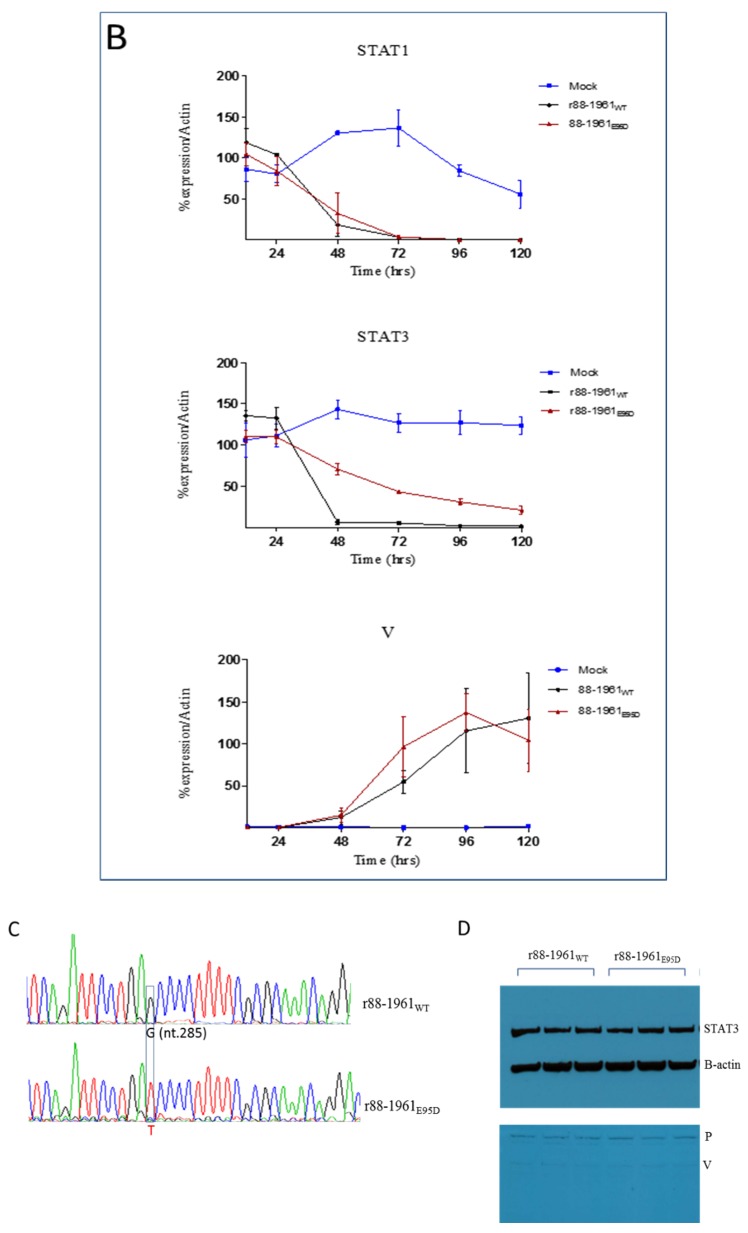
Degradation of STAT proteins in vitro and in vivo (r88-1961_WT_ and r88-1961_E95D_). (**A**) Confluent monolayers of Vero cells were infected with r88-1961_WT_ and r88-1961_E95D_ at an MOI of 1.0. Cell lysates prepared at the times indicated were subjected to SDS-PAGE and Western blot. The blots were probed with anti-STAT1, anti-STAT3, anti-B-actin, and anti-mumps P/V antibodies. Although no significant differences were observed in the degradation of STAT1, degradation of STAT3 was noticeably delayed in cells infected with r88-1961_E95D_ versus those infected with r88-1961_WT_. Reduced V_E95D_ activity corresponded with a concomitant increase in V_E95D_ versus V_WT_ expression. (**B**) To quantify the data shown in the Western blots in panel (A), a series of Western blots were performed from lysates collected longitudinally. The expression kinetics of STAT1, STAT3 and V were evaluated in three independent experiments and band intensities quantified using the Image J program. The data represents mean values ± standard error of the mean and reaffirm the findings observed in the individual blot. (**C**) Maintenance of the wild type V gene nucleotide (G) in r88-1961_WT_ and the presence of the engineered substitution (T) in r88-1961_E95D_ was confirmed in Vero cells infected with r88-1961_WT_ and r88-1961_E95D_ at 120 h post inoculation. The sequence was confirmed independently in triplicate infections per virus variant. (**D**) Newborn rats inoculated intracerebrally in triplicate with r88-1961_WT_ and r88-1961_E95D_ were euthanized on day 3 post inoculation. The brain lysates were subjected to SDS-PAGE and western blot. The blots were probed with anti-STAT3 and anti-B-actin (top panel) and anti-P/V (bottom panel) antibodies. Given that only 1% of brain cells are infected by MuV, no differences were observed in STAT3 levels in vivo between rats infected with r88-1961_E95D_ versus r88-1961_WT_. Note, P/V bands are present, but faint (bottom panel, all lanes).

**Figure 4:**
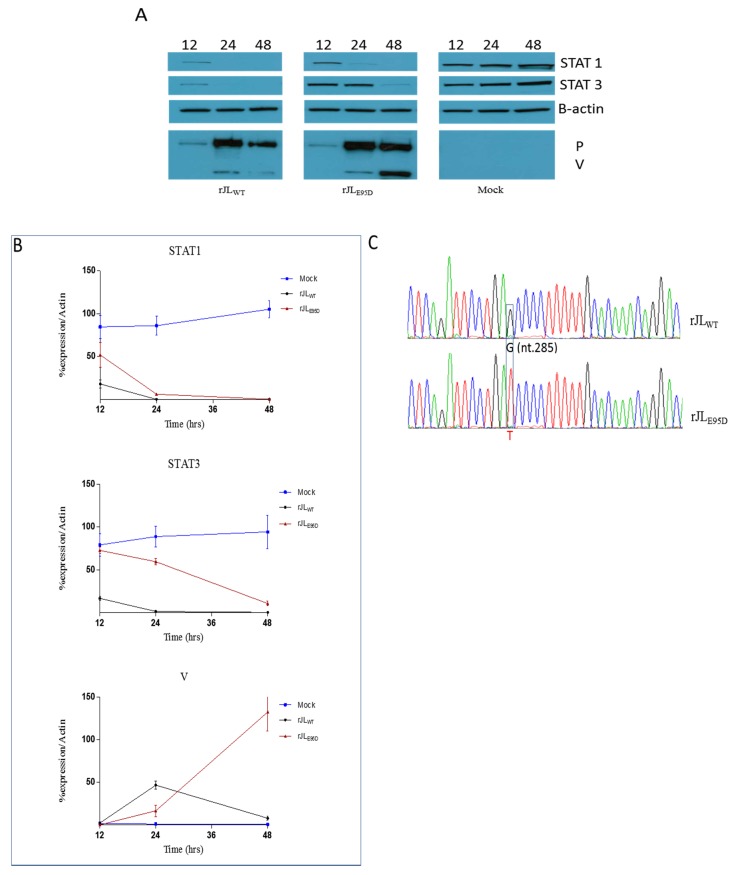
Degradation of STAT proteins in vitro (rJL_WT_ and rJL_E95D_). (**A**) Confluent monolayers of Vero cells were infected with rJL_WT_ and rJL_E95D_ at an MOI of 1.0. Cell lysates prepared at the times indicated were subjected to SDS-PAGE and Western blot. The blots were probed with anti-STAT1, anti-STAT3, anti-B-actin, and anti-mumps P/V antibodies. Although delayed, STAT1 was completely degraded by 48 h P.I. in cells infected with rJL_E95D_ versus those infected with rJL_WT_. Degradation of STAT3 was, however, significantly delayed in cells infected with rJL_E95D_ versus those infected with rJL_WT_. Reduced V_E95D_ activity corresponded with a concomitant increase in V_E95D_ versus V_WT_ expression. (**B**) To quantify the data shown in the Western blots in panel (A), a series of Western blots were performed from lysates collected longitudinally. The expression kinetics of STAT1, STAT3 and V were evaluated in three independent experiments and band intensities quantified using the Image J program. The data represents mean values ± standard error of the mean and reaffirm the findings observed in the individual blot. (**C**) Maintenance of the wild type V gene nucleotide (G) in rJL_WT_ and the presence of the engineered substitution (T) in rJL_E95D_ was confirmed in Vero cells infected with rJL_WT_ and rJL_E95D_ at 48 h post inoculation. The sequence was confirmed independently in triplicate infections per virus variant.

The MuV V protein has been shown to target STAT3 for degradation [28,32,34] and that an E95D amino acid substitution in the protein inhibits this activity [34]. Here we confirm these findings, but also show that the E95D substitution does not completely abrogate V protein ability to degrade STAT3, as indicated by the gradual decline in STAT3 levels over a five-day period after inoculation with r88-1961_E95D_ (Figure 3a,b, respectively). Similar results were also obtained upon infection of FADU cells with the r88-1961_WT_ and mutant V MuV’s, respectively (Supplemental Figure S1). Importantly, this result was also observed with rJL_E95D_ infected cells (Figure 4a,b). It should be noted that given the extreme lytic nature of Jeryl Lynn, the kinetics of degradation could only be followed for up to 48 h P.I. The E95D substitution did not alter the ability of either the 88-1961 or Jeryl Lynn derived V protein to completely degrade STAT1 (Figure 3a,b and Figure 4a,b), although we did consistently observe a slower degradation of STAT1 in rJL_E95D_ infected cells. In each case, the reduced ability to degrade STAT3 does not appear to be due to a reduction in V protein levels. In fact, we observed elevated levels of V_E95D_ in r88-1961_E95D_ and rJL_E95D_ infected cells (quantified longitudinally in Figure 3b and Figure 4b, respectively). Sequencing of r88-1961_E95D_ and rJL_E95D_ infected cells from later time points demonstrate maintenance of the E95D mutation, indicating that the gradual decrease in STAT3 levels is not due to reversion of V_E95D_ to wild type V (Figure 3c and Figure 4c). Notably, it is not possible to evaluate virus-specific effects on STAT3 levels in rat brain using this method given that any effect on STAT3 in the relatively few infected cells (<1%) would be overshadowed by normal levels of STAT3 in the overwhelming number of uninfected cells in the brain. Consequently, as expected, no differences were observed in STAT3 levels in vivo between rats infected with r88-1961_E95D_ versus r88-1961_WT_ (Figure 3d).

### 3.5. Relative Binding Affinity of the V Protein Variants to Cellular Partners

**Figure 5:**
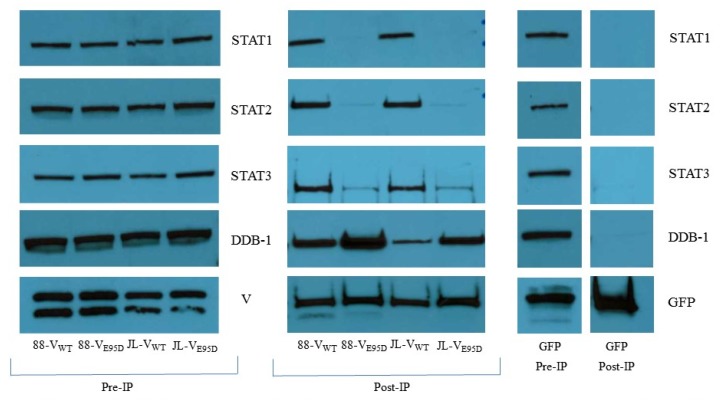
Association of the MuV V protein variants with cellular proteins. Confluent monolayers of 293-A cells were transfected with FLAG-tagged V or FLAG-tagged GFP expressing constructs and harvested 48 h post-transfection. The cells were processed for affinity purification with a fraction reserved for direct lysis in sample buffer (pre-IP). Protein complexes bound to the anti-FLAG agarose beads were eluted in sample buffer (post-IP). Samples were resolved by SDS-PAGE followed by western blotting. The blots were probed with anti-STAT1, anti-STAT2, anti-STAT3, anti-DDB-1, anti-GFP and anti-mumps P/V antibodies. Note, this blot is representative of three independent experimental repeats. While the 88-1961 and JL derived V_E95D_ proteins coprecipitated STAT1, 2 and 3 extremely weakly (faint bands were consistently present for the three proteins in each repeat experiment) compared to V_WT_, increased coprecipitation of DDB1 was observed relative to the wild type V counterpart. The pre-IP blot in each case demonstrates equal amounts of lysates were used for the pulldown, and the FLAG-Tagged GFP pulldown demonstrates the specificity of the assay.

The mumps V protein has been shown to interact with a complex of proteins, including the STAT proteins and host factors, such as DDB1 [32,34], in order to target STAT1 and STAT3 for degradation. The 88-1961 and Jeryl Lynn derived wild type and E95D V variant proteins were assessed, through co-immunoprecipitation studies with FLAG-tagged V protein, to determine if decreased ability to degrade STAT3 correlated with decreased binding to V. In each experiment, cells transfected with a FLAG-tagged GFP expressing plasmid were used as control to confirm the specificity of the interactions observed. Significantly less STAT3 coprecipitated with either of the E95D mutant V proteins in comparison to the amount of STAT3 that coprecipitated with the wild type counterparts (Figure 5). Unexpectedly, we also observed extremely reduced coprecipitation of both STAT1 and STAT2 with the 88-1961 and Jeryl Lynn derived E95D V proteins relative to the wild type counterparts (Figure 5). Interestingly, however, both the 88-1961 and Jeryl Lynn derived E95D V proteins coprecipitated with DDB1 at very high levels relative to the wild type derived V proteins (Figure 5) suggesting that V_E95D_ may be able to bind more molecules of DDB1, due to increased availability of unbound V protein, arising from it’s inability to bind the STAT proteins effectively. 

## 4. Discussion

Most viruses that infect eukaryote cells have evolved mechanisms to circumvent innate antiviral responses in the infected host. The paramyxovirus V proteins are one example. These proteins interact with a number of cellular proteins involved in the innate antiviral response, including STAT proteins. Upon virus infection, numerous cellular factors, including interferons, can activate STAT proteins, which in turn bind to the promoters of a vast array of genes, including those encoding proteins with antiviral activities, as well as proteins that negatively regulate the antiviral response. The V protein of some paramyxoviruses (e.g., parainfluenza virus 5 (PIV5) and human parainfluenza virus 2) target STAT1 and STAT2 for proteasome-mediated degradation [39,40,41,42], whereas, others (e.g., Tioman virus and MuV) target STAT1 and/or STAT3 for degradation [28,34,42,43]. For PIV5, a virus in the same genus as MuV (Rubulavirus), mutations at amino acid position 100 of the V protein were shown to significantly affect the formation of V protein-dependent degradation complexes with STAT1 and STAT2. Whereas, an N100D substitution in SV5 V conferred the ability to block interferon signaling in murine cells through STAT 1 degradation [44], an N100R substitution abolished the ability of the V protein to interact with STAT 2 [45]. Based on sequence alignment, amino acid position 100 in the PIV5 V protein is analogous to position 95 in the MuV V protein, thus Puri et al. made the two corresponding mutants (E95D and E95R) in MuV using the attenuated Jeryl Lynn MuV vaccine strain and evaluated the effect on STAT levels [34]. While the E95D mutation had no effect on the ability of the virus to target STAT1 for degradation, it abolished the ability of the virus to target STAT3 for degradation. Similar results were obtained with the E95R MuV V protein mutant, but the effect was less pronounced. Prevention of V protein expression significantly attenuates MuV in vivo (28); however, given that this protein targets both STAT1 and STAT3 for degradation, as well as possibly having other roles, the mechanism of the attenuation is not clear. The ability of the E95D mutation to disrupt targeting of STAT3, but not STAT1, afforded us the opportunity to examine the role of STAT3 in the virulence of a wild type recombinant MuV strain. While virulence could not be examined for Jeryl Lynn given that this strain is fully attenuated, we did perform in-vitro studies using JL_WT_, JL_E95D_ and the corresponding wild type and mutant V protein’s to corroborate our in vitro findings for 88-1961. It should be noted that position 95 is located in the common N-terminal domain shared by the P and V proteins, and as such the modification also resulted in an amino acid change in P. However, this change did not appear to diminish expression levels of P as demonstrated by western blot (Figure 3a and Figure 4a), nor P protein function as suggested by our ability to efficiently rescue the virus following transfection and given the very similar kinetics of virus replication in vitro (Figure 1). Importantly, Xu et al. [28] and Kubota et al. [30] have each demonstrated that it is the mumps V, and not P, that plays the key role in the degradation of the STAT proteins.

Here we confirmed the finding by Puri et al. that the E95D mutation in the V protein inhibits MuV targeting of STAT3 for degradation when assessed 48 h post-infection [34], however, when observation was continued to later time points, it was clear that the E95D substitution does not completely abrogate V protein ability to degrade STAT3 and that STAT3 levels were nearly as low as those seen in cultures infected with the parental wild type virus. Our data demonstrate that the reduced ability of V_E95D_ to target STAT3 for degradation correlates not only with reduced coprecipitation with STAT3, but also with increased expression of the mutant V protein in r88-1961_E95D_ or rJL_E95D_ infected cells. The increase in V_E95D_ expression over-time may be the consequence of a feedback mechanism where the virus is attempting to compensate for the lack of V protein activity. Whether or not such feedback loops exist is not known. 

It should be noted that although Puri et al. reported the inability of JL-V_E95D_ to bind to STAT3 [34], we consistently observed coprecipitation of 88-1961-V_E95D_ and JL-V_E95D_ with STAT3 over multiple independent experimental repeats, albeit at significantly reduced levels relative to the wild type V counterparts.

Introduction of the E95D mutation in the JL-V protein has been reported not to impact the degradation of (examined at 48 h P.I. only) nor binding to STAT1 [34]. While this was confirmed in our studies of 88-1961-V_E95D_, there was a noticeable delay in degradation of STAT1 by JL-V_E95D_ versus JL-V_WT_ (although, both JL-based viruses completely degraded STAT1 by 48 h P.I.). More surprising was that although 88-1961-V_WT_ and JL-V_WT_ bound to STAT1 based on pulldown experiments, neither 88-1961-V_E95D_ nor JL-V_E95D_ could coprecipitate STAT1 to any significant extent. This finding is consistent with our observation that the V_E95D_ variant proteins also displayed dramatically reduced coprecipitation with STAT2. Multiple studies [40,46,47] have demonstrated an interrelationship between the binding of STAT1 and STAT2 to rubulavirus V proteins. In contrast, Puri et al. reported no differences in the binding of JL-V_WT_ versus JL-V_E95D_ to STAT1, despite observing reduced coprecipitation of JL-V_E95D_ with STAT2. Based on our findings, we postulate that despite the markedly reduced coprecipitation of STAT1 and STAT2 with V_E95D_, binding between these proteins is sufficient to effectively target STAT1 for degradation and/or that increased V_E95D_ expression relative to V_WT_ can compensate for the diminished V binding activity. It is worth noting that the V protein binds STAT1 and STAT3 independently and as such it was not entirely unexpected that the degradation of these proteins is impacted differently despite reduced binding of both STAT1 and STAT3 to V_E95D_.

The paramyxovirus V complexes (VDC’s) that direct the degradation of the STAT1 and STAT3 proteins require the recruitment of several cellular partners, including DDB1, Cul4A and the E3 enzymes [32,33,41,45,46,48]. In particular, all rubulavirus V proteins examined, thus, far require the recruitment of DDB1 to enable STAT protein degradation [32,33,41,49]. Here, in agreement with Puri et al., we have shown a physical association between DDB1 and both the wild type and E95D variant V proteins derived from either 88-1961 or Jeryl Lynn. However, unlike the prior report, we consistently observed more DDB1 bound to the E95D variant proteins. Given the dramatically reduced ability of the mutant V variants to coprecipitate the STAT proteins, we postulate that this finding may reflect the availability of unbound V protein to recruit additional molecules of DDB1. It would be interesting to determine if this finding holds true for the other cellular partners required for the degradation complex.

Despite the significant impact of the E95D mutation on the STAT pathways in vitro, r88-1961_E95D_ displayed similar replication kinetics to the wild type virus at both low and high MOIs. This effect was observed in both non-IFN producing cells (Vero) and in IFN producing cells (FaDu), suggesting that our data are not an artefact of using cells incapable of producing IFN. Similarly, no differences have been reported between the in vitro growth kinetics of the rJL_WT_ and rJL_E95D_ variant viruses [34]. In contrast, in vivo, the r88-1961_E95D_ virus was partially attenuated (resulting in less severe hydrocephalus) in comparison to the parental wild type genotype. The observed clinical attenuation correlated with r88-1961_E95D_ growing to lower levels and being cleared faster from the brain than the wild type virus. It should be noted that the partial functionality of the mutant V demonstrated in vitro correlates with the partial attenuation observed in vivo. While we did not find evidence of an effect of the E95D mutation on total STAT3 levels in rat brain, this was expected in that only a small fraction (far less than 1%) of brain cells are infected by MuV [50], thus, any effect of the viral V protein on STAT3 levels would be overshadowed by steady state expression levels of STAT3 in the rest of the brain. 

As to the discordance between the in vitro and the in vivo growth kinetics, this is difficult to explain; however, it is important to point out that the activation of STAT3 has been found to have a very wide range of effects, which seem to be cell-specific. Some have reported STAT3 activation to promote antiviral activity [32,34,43], some have reported STAT3 activation to inhibit antiviral activity [51,52,53], and still, others have found that STAT3 both inhibits and promotes antiviral activity [54]. Given that STAT3 forms heterodimers with other STATs [55] and given the wide array of molecules that activate STAT3 and its role in mediating signaling of diverse pathways [32,34,39,43,51,52,53,54], these seemingly inconsistent findings between the in vitro and in vivo environments are perhaps not surprising. In our study, inhibition of virus-mediated degradation of STAT3 leads to more rapid viral clearance, suggesting that at least in the context of MuV infections, STAT3 is a mediator of antiviral responses. 

In summary, we have shown here that a single amino acid substitution in the mumps virus V protein alters the ability of the protein to target STAT3 for degradation in a more subtle manner than reported previously. We have also shown the effects of the E95D mutation impact binding with STAT1 and STAT2, while degradation of these proteins remains largely unaltered. Finally, this single mutation is potent enough not to allow MuV to effectively evade the host immune system as demonstrated by faster clearance and partial neuroattenuation of the virus in rat brain. Given the renewed interest in the development of more effective safe mumps vaccines, these observations may also be of practical relevance. Indeed, approaches to virus attenuation are gradually maturing from the historical methods of empiric attenuation through blind, hopeful passaging in unnatural culture systems to more rational, directed manipulations using reverse genetics.

## Supplementary Materials

The following are available online at https://www.mdpi.com/1999-4915/11/11/1016/s1, Figure S1: Degradation of STAT3 in FADU Cells. Confluent monolayers of FADU cells were infected with r88-1961_WT_ and r88-1961_E95D_ at an MOI of 1.0. Cell lysates prepared at the times indicated were subjected to SDS-PAGE and Western blot. The blots were probed with anti-STAT3, anti-B-actin, and anti-mumps P/V antibodies. Degradation of STAT3 was noticeably delayed in cells infected with r88-1961_E95D_ versus those infected with r88-1961_WT_. Reduced VE95D activity corresponded with a concomitant increase in V_E95D_ versus V_WT_ expression.
