# Supplementary material for: A Single Point Mutation in the Mumps V Protein Alters Targeting of the Cellular STAT Pathways Resulting in Virus Attenuation
Source: Viruses. 2019 Nov 1;11(11):1016. doi: 10.3390/v11111016 (PMC6893744; doi:10.3390/v11111016)
Supplement: Supplementary file 1 [file viruses-11-01016-s001.pdf]

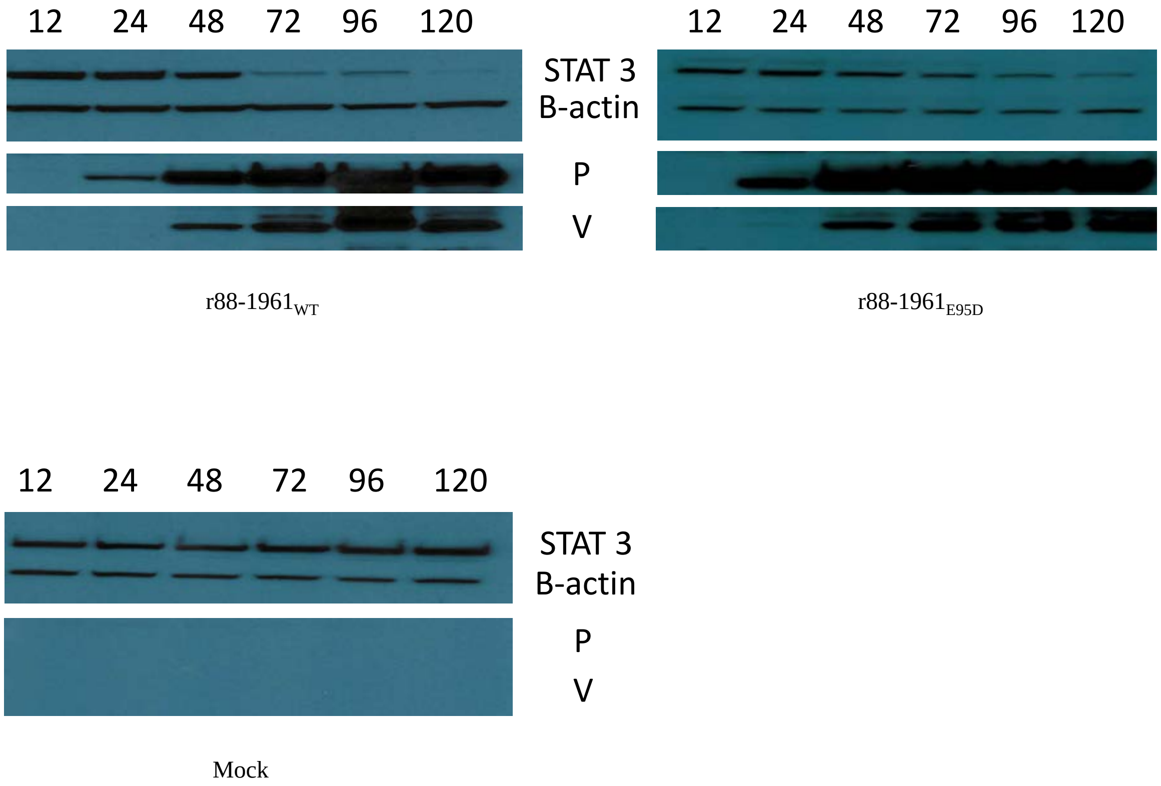

Supplemental Figure 1. Degradation of STAT 3 in FADU Cells. Confluent monolayers of FADU cells were infected with r88-1961$_{WT}$ and r88-1961$_{E95D}$ at an MOI of 1.0. Cell lysates prepared at the times indicated were subjected to SDS-PAGE and Western blot. The blots were probed with anti-STAT3, anti-B-actin, and anti-mumps P/V antibodies. Degradation of STAT3 was noticeably delayed in cells infected with r88-1961$_{E95D}$ versus those infected with r88-1961$_{WT}$. Reduced V$_{E95D}$ activity corresponded with a concomitant increase in V$_{E95D}$ versus V$_{WT}$ expression.
